# Hypertrophy of Nails and Bone

**Published:** 1869-10

**Authors:** 


HYPERTROPHY OF NAILS AND BONE.—In *Virchow's Archiv* is recorded by Professor Friedrich, of Heidelberg, the case of a shoemaker, 26 years of age, in whom the nails had grown to a prodigious size, those of the middle finger measuring nearly an inch, while that of the thumb was a little less, and the nail of the great toe somewhat more than an inch and a half in length. Eight years back he observed an increase of size of one foot; the enlargement extended to the leg, and two years later the same remarkable growth appeared in his hands. On examination, the enlargement was seen to be referable to the bones, and by degrees every bone in the body became similarly affected. The teeth escaped change, as did the tracha, but the cartilages of the pinna and the epiglottis were greatly enlarged. His skin was somewhat indurated, and the muscles soft and flabby. A brother, aged 22, exhibited a similar tendency to hyperostosis, which had commenced four years previously. Reference is also made to a case mentioned by *Sancerette* of a man who had increased in weight fifty-nine pounds from the mere growth of his bones in bulk, the soft parts, in the meantime, having become considerably wasted.

